# Supplementary material for: Effective lifting of the topological protection of quantum spin Hall edge states by edge coupling
Source: Nat Commun. 2022 Jun 16;13:3480. doi: 10.1038/s41467-022-30996-z (PMC9203811; doi:10.1038/s41467-022-30996-z)
Supplement: Supplementary file 1 — Supplementary Information [file 41467_2022_30996_MOESM1_ESM.pdf]

# Supplementary Information

—

# Effective lifting of the topological protection of quantum spin Hall edge states by edge coupling


R. Stühler,[1,*] A. Kowalewski,[1] F. Reis,[1] D. Jungblut,[2] F. Dominguez,[2,3]
B. Scharf,[2] G. Li,[2,4,5] J. Schäfer,[1] E. M. Hankiewicz,[2] and R. Claessen[1]

[1]*Physikalisches Institut and Würzburg-Dresden Cluster of Excellence ct.qmat, Universität Würzburg, D-97074 Würzburg, Germany*
[2]*Institut für Theoretische Physik und Astrophysik and Würzburg-Dresden Cluster of Excellence ct.qmat, Universität Würzburg, D-97074 Würzburg, Germany*
[3]*Institute for Mathematical Physics, TU Braunschweig, 38106 Braunschweig, Germany*
[4]*School of Physical Science and Technology, ShanghaiTech University, Shanghai 201210, China*
[5]*ShanghaiTech Laboratory for Topological Physics, Shanghai 200031, China*



(Dated: May 4, 2022)



* raul.stuehler@physik.uni-wuerzburg.de

## CONTENTS

A. DB measured with 'sharp' tip configuration 1
B. Metallic edge state spreads along circumference of exposed zigzag edge 2
C. Domain boundary different length 3
D. Variation of Valence Band Onset 4
E. Isolated zigzag edge and mirroring procedure 5
F. Tight binding Hamiltonian of Bismuthene on SiC 6
1. Topological invariant 7
2. Line defect 8
G. Fabry-Pérot states in different domain boundaries 11
H. 1/L dependence of Fabry-Pérot resonances 15
I. Coupled Fabry-Pérot resonators 16

References 18

### A. DB measured with 'sharp' tip configuration

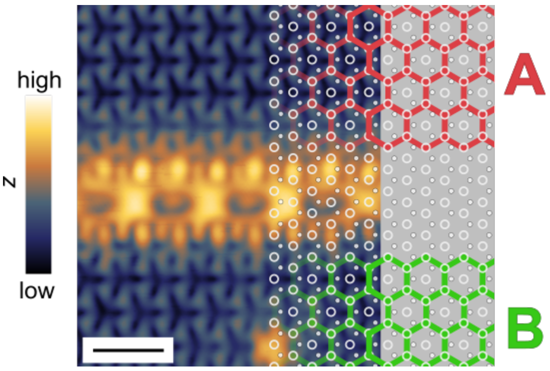


FIG. S1. Constant-current STM measurement of a DB measured with different tip preparation, i.e. poaching the tip several Å into the bismuthene surface. The topography of the bismuth atoms on honeycomb sites appears much sharper compared to a STM tip prepared on Ag(111). The pronounced $C_3$ rotational symmetry about the honeycomb center stems from the $C_3$ symmetry of the SiC(0001) substrate, i.e. induced by the carbon layer. With this tip configuration the maximum separation distance between two bismuthene zigzig edges at domains 'A' and 'B' adjacent to the DB is clearly seen to be 15 Å. The schematic atomic lattice indicates the SiC(0001) structure with open circles representing Si atoms and small dots representing C atoms. The bismuthene lattice is indicated as a red and green honeycomb lattice. Scan parameters: $V_{\text{set}} = -1.0\,\text{V}$, $I_{\text{set}} = 300\,\text{pA}$. Scale bar: 1 nm

## B. Metallic edge state spreads along circumference of exposed zigzag edge

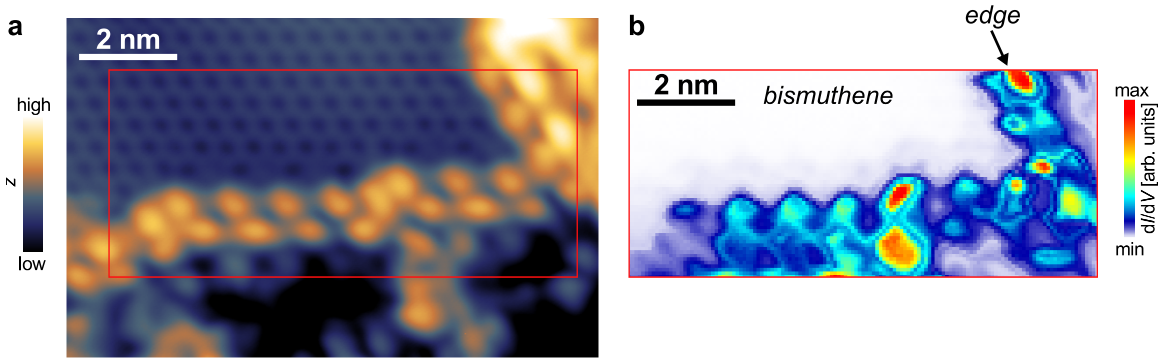


FIG. S2. **a**, Constant-current STM measurement of the free zigzag edge in Fig. 3b. Scan parameters: $V_{\text{set}} = -1.2\,\text{V}$, $I_{\text{set}} = 20\,\text{pA}$. **b**, Constant-energy $\mathrm{d}I/\mathrm{d}V$ map within the bulk band gap of bismuthene at $E - E_{\text{F}} = 0.422\,\text{eV}$ (averaged over $\pm 20\,\text{mV}$). The region for the spatially resolved $\mathrm{d}I/\mathrm{d}V$ mapping is indicated by the red rectangle in **a**. This indicated that metallic LDOS spreads along the edge as a 1D-channel.

### C. Domain boundary different length

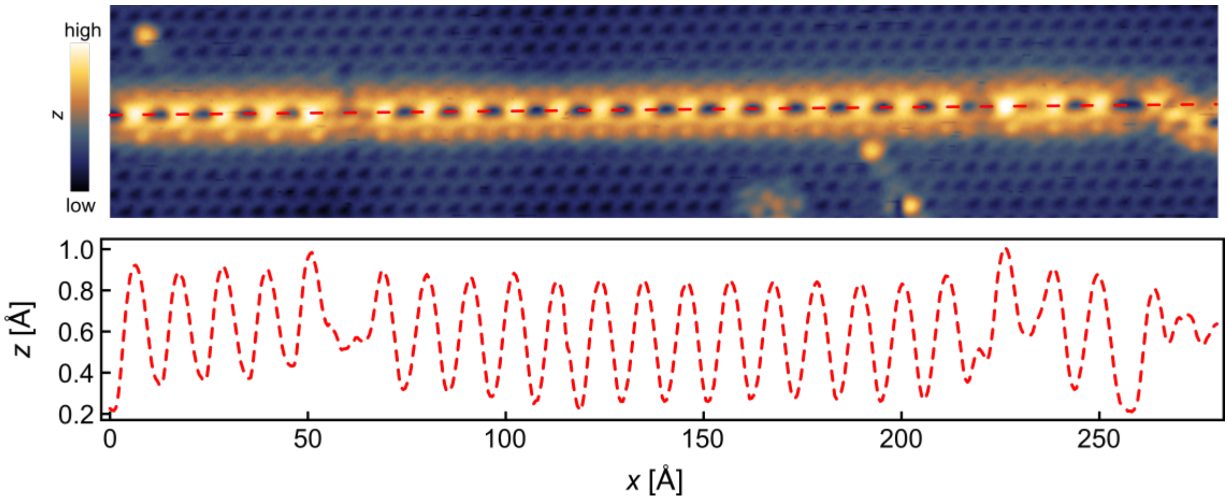


FIG. S3. Upper panel: Constant-current STM measurement of a DB with a lengths of $\sim 25\,\mathrm{nm}$. Scan parameters: $V_{\mathrm{set}} = -1.2\,\mathrm{V}$, $I_{\mathrm{set}} = 40\,\mathrm{pA}$. Lower panel: height profile along the red dashed line in the upper panel.

### D. Variation of Valence Band Onset

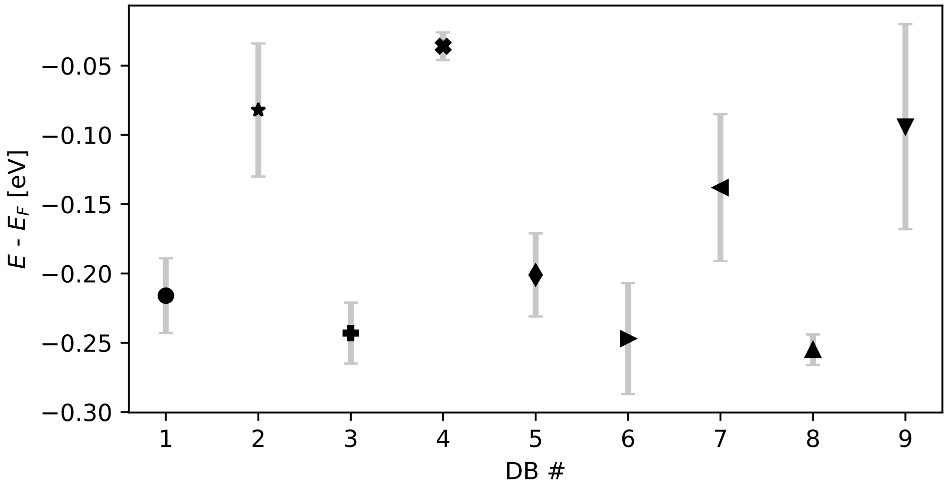


FIG. S4. Valence band onset measured at the vicinity of each DB 1 – 9. The valence band onsets account for a local variation of the local chemical potential $\mu$, which are found on the bismuthene surface. The error bars indicate the standard deviation (s.d.) from the mean values in an area around each DB. The valence band onsets are used to consistently define an energy onset between all DBs. The data points are assigned marker symbols according to the marker symbols for each DB used in Fig. 4a.

### E. Isolated zigzag edge and mirroring procedure

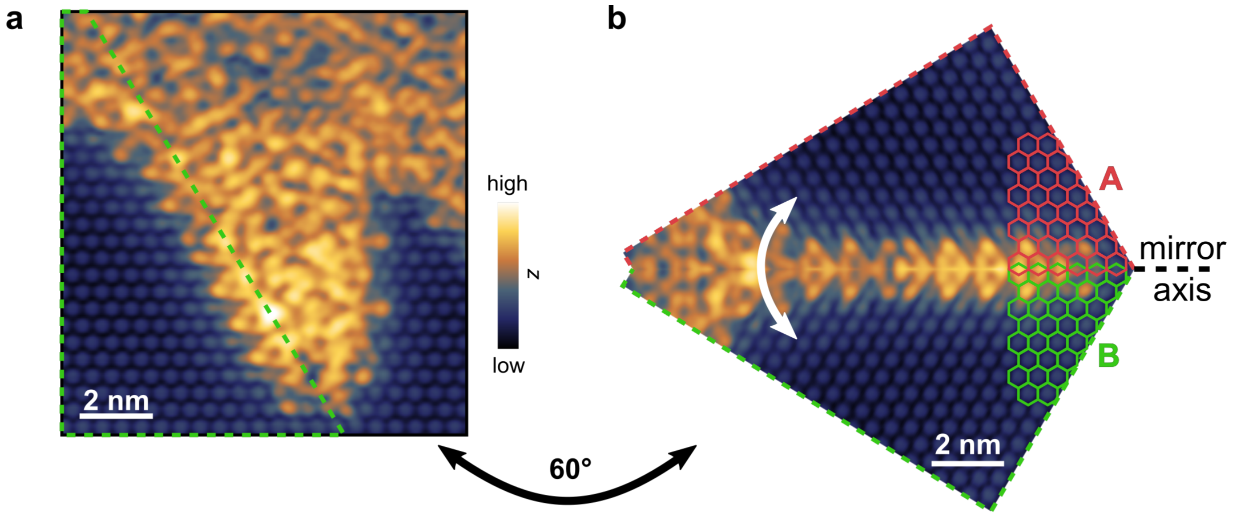


FIG. S5. **a**, STM constant-current map showing a free zigzag edge of bismuthene. **b**, The image is the result of: 1. extracting the region marked with a green-dashed line in **a**; 2. rotating it by 60° ; 3. mirroring at the marked mirror axis such that domain 'A' is mirrored into domain 'B'. This operation results in an image that properly generates an adsorption site shift to mimic the situation at a DB. The registry mismatch between both domains can be seen in the respective hexagonal lattices. Scan parameters for **a, b**: $V_{\mathrm{set}} = 1.6\,\mathrm{V}, I_{\mathrm{set}} = 70\,\mathrm{pA}$ .

## F. Tight binding Hamiltonian of Bismuthene on SiC

Bismuthene on silicon carbide (Bi/SiC) is a 2-dimensional hexagonal lattice material which exhibits a large topological bulk energy-gap [S1]. The large size of the topological gap $\sim 0.8\,\mathrm{eV}$ opened at the - and $K'$-points is attributed to the intrinsic on-site spin-orbit coupling (SOC), which owes its strength to an orbital-filtering mechanism mediated by the SiC substrate: the coupling to the SiC substrate shifts energetically the $p_z$-weighted orbitals leaving only $p_x$- and $p_y$-orbitals in the low-energy sector, which have a stronger coupling. Using DFT calculations and a standard "down folding" procedure of the $s$- and $p_z$-orbitals it is possible to describe effectively the Bi/SiC low-energy Hamiltonian by means of an 8×8 $\sigma$-band Hamiltonian [S2, S3]. Here, we write the spatial representation, containing the tunnel amplitudes along the $t_1, t_2, t_3$ directions, see Fig. S6a.

$$H_{\mathrm{eff}}^{t_i} = H_0^{t_i} + \lambda_{\mathrm{SOC}} H_{\mathrm{SOC}}^{t_i} + \lambda_{\mathrm{R}} H_{\mathrm{R}}^{t_i} + \lambda_{\mathrm{V}} H_{\mathrm{V}}^{t_i}, \tag{S1}$$

which accounts for the coupling between the sublattice, $p_x - p_y$ orbitals and spin degrees of freedom, with the corresponding subspaces spanned by the Pauli matrices $\sigma$, $\pi$ and $s$, respectively. This model is further used to describe other 2-dimensional hexagonal lattice topological insulator materials with two orbitals, such as antimonene (Sb/SiC) and arsenicene (As/SiC) [S3]. The first term on the right-hand side of Eq. (S1) accounts for the spin-independent hopping between the $p_x$ and $p_y$ orbitals of nearest neighbour atoms along the $t_i$ direction and is given by

$$H_0^{t_i} = \frac{1}{4}\sigma_+ \otimes s_0 \otimes \tilde{H}_0^{t_i} + \mathrm{h.c.}, \tag{S2}$$

with $\sigma_\pm = (\sigma_1 \pm i\sigma_2)/2$ and

$$\begin{aligned}
\tilde{H}_0^{t_1} &= 2\left[(\pi_0 + \pi_3)V_{pp\pi} + (\pi_0 - \pi_3)V_{pp\sigma}\right],\\
\tilde{H}_0^{t_2} &= \left[(\pi_3 + \pi_1\sqrt{3})(V_{pp\sigma} - V_{pp\pi}) + 2\pi_0(V_{pp\pi} + V_{pp\sigma})\right],\\
\tilde{H}_0^{t_3} &= \left[(\pi_3 - \pi_1\sqrt{3})(V_{pp\sigma} - V_{pp\pi}) + 2\pi_0(V_{pp\pi} + V_{pp\sigma})\right].
\end{aligned}$$

Here, $V_{pp\sigma}$ and $V_{pp\pi}$ are the overlap integrals between $p_x/p_y$-orbitals placed on different sublattices. The term $H_{\mathrm{SOC}}$ provides the intrinsic SOC, which opens a gap of $2\lambda_{\mathrm{SOC}} = 0.87\,\mathrm{eV}$ at the $K$ and $K'$-points and it is given by

$$H_{\mathrm{SOC}}^{t_i} = \sigma_0 \otimes s_3 \otimes \pi_2. \tag{S3}$$

The Rashba-type SOC accounts for the coupling between the SiC-substrate and the hexagonal Bi-monolayer, where the substrate acts like a static electric field $E$ with coupling strength $\lambda_{\mathrm{E}}$ pointing out of plane. In Eq. (S1), these effects can be described by the effective $\sigma$-band model $H_{\mathrm{R}}^{t_i} = (i/2)\sigma_+ \otimes \tilde{H}_R^{t_i} + \mathrm{h.c.}$ where

$$\begin{aligned}
\tilde{H}_R^{t_1} &= 2\left[s_1 \otimes (\pi_3 - \pi_0) + s_2 \otimes \pi_1\right],\\
\tilde{H}_R^{t_2/t_3} &= \left[s_1 \otimes (\pm\sqrt{3}\pi_1 + \pi_0 - \pi_3) \mp \sqrt{3}s_2 \otimes (\pi_0 + \pi_3 \pm \frac{\pi_1}{\sqrt{3}})\right].
\end{aligned} \tag{S4}$$

Moreover, $\lambda_{\mathrm{R}}$ in Eq. (S1) is given by $\lambda_{\mathrm{R}} = \lambda_{\mathrm{SOC}} V_{sp\sigma}/\lambda_{\mathrm{E}}$. The Rashba-type SOC breaks the particle-hole symmetry (PHS) of the Hamiltonian resulting in a valence band splitting of $12\lambda_{\mathrm{R}}$ with $\lambda_{\mathrm{R}} = 0.032\,\mathrm{eV}$, see Fig. S6b.

Finally, we introduce ad-hoc the last term in Eq. (S1) to fit the experimental observations. Note that this term does not change the physics of the model nor its topological properties. It only allows to tune the position of the Dirac cone and the Fermi velocity $v_{\mathrm{F}}$ of the edge states. Since the Hamiltonian has initially been derived to fit DFT calculations around the $K$- and $K'$-points, it is natural to assume that the parameters can change around the $\Gamma$ point. Thus, we have added the simplest term that preserves time-reversal symmetry but breaks particle-hole symmetry, that is

$$H_{\mathrm{V}}^{t_i} = \sigma_0 \otimes s_0 \otimes \pi_3. \tag{S5}$$

In contrast to the Rashba Hamiltonian, this term does not mix the spin degrees of freedom.

*Periodic boundary conditions (PBC)*— Once we have established the spatial representation of the hexagonal lattice Hamiltonian, we define a flake structure comparable to the scheme shown in Fig. S7c. Subsequently, we impose periodic boundary conditions in both directions and add a Bloch phase in the tunneling amplitudes that couple

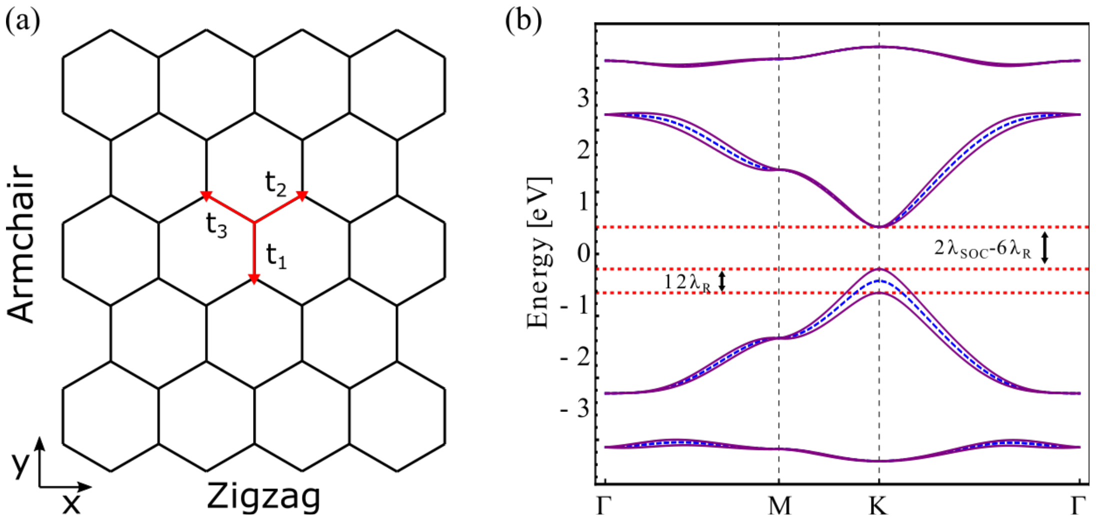


FIG. S6. Panel (a) hexagonal lattice of Bi/SiC with armchair and zigzag boundary conditions. We mark with red arrows the nearest neighbour hopping directions $t_1, t_2, t_3$. Panel (b) shows the bulk energy dispersion along the high symmetry momenta using the parameters fitted around the $K/K'$-points $\lambda_{\rm SOC} = 0.435\,{\rm eV}$, $\lambda_{\rm R} = 0.032\,{\rm eV}$, $V_{sp\sigma} = 2.0\,{\rm eV}$ and $V_{sp\pi} = 0.21\,{\rm eV}$. The horizontal red dotted lines mark the valence band splitting due to the Rashba spin-orbit coupling ($12\lambda_{\rm R}$) and the bulk energy gap ($12\lambda_{\rm SOC} - 6\lambda_{\rm R}$) at the $K$-point.

different sublattices, that is, $\sigma_\pm \to e^{\pm i/2 f_{t_i}(k_x,ky)a}\sigma_\pm$, with $a = 5.35\,\text{Å}$, being the Bi/SiC lattice constant and the phases

$$f_{t_1}(k_x, k_y) = 0, \tag{S6}$$

$$f_{t_2}(k_x, k_y) = \sqrt{3}k_y + k_x, \tag{S7}$$

$$f_{t_3}(k_x, k_y) = \sqrt{3}k_y - k_x. \tag{S8}$$

With these replacements, one can obtain the bulk Hamiltonian $H_{bulk}(k_x, k_y)$ presented in Refs. [S1–S3]

### 1. *Topological invariant*

Here, we calculate the topological invariant in the presence of both time-reversal symmetry $\mathcal{T}$ and inversion symmetry $\mathcal{I}$. To do so, we need to neglect the Rashba spin-orbit contribution ($\lambda_{\rm R} = 0$) since it breaks $\mathcal{I}$. For the typical values estimated in Bi/SiC, its presence does not modify the topological regions determined by the inversion symmetric Hamiltonian. In this way, the topological invariant can be calculated in a very simple way by expressing the bulk Hamiltonian in the basis which diagonalizes the inversion symmetry operator $\mathcal{I} = \sigma_1 \otimes \pi_0 \otimes s_0$ [S4]. The resulting matrix is block diagonal at the time reversal invariant momenta (TRIM) points since $[H_{bulk}(\Gamma_i), \mathcal{I}] = 0$. These blocks exhibit a different parity eigenvalue at the TRIM $\xi_{2m}(\Gamma_i) = \pm 1$ and the topological invariant $n_{z_2}$ is given as a product of these parity eigenvalues, namely

$$(-1)^{n_{z_2}} = \prod_{i=TRIM} \delta_i, \tag{S9}$$

$$\delta_i = \prod_{m=1}^{n} \xi_{2m}(\Gamma_i), \tag{S10}$$

Here, the product only accounts for the half of the Kramers partners from the $n$ occupied states.

The TRIM are placed at $\Gamma_1 = (0, 0)$, $\Gamma_2 = \frac{2\pi}{a}(0, \frac{1}{\sqrt{3}})$, $\Gamma_3 = \frac{2\pi}{a}(\frac{1}{2}, -\frac{1}{2\sqrt{3}})$,$\Gamma_4 = \frac{2\pi}{a}(-\frac{1}{2}, -\frac{1}{2\sqrt{3}})$, which correspond to the $\Gamma$-point, the $M$-point and the $K$-points, respectively. Substituting the TRIM into Eq. (S10), we find that $\delta_3 = \delta_4$,

and therefore, the topological invariant is entirely determined by the product of

$$\delta_1 = \text{Sign}\left[\frac{9}{4}(V_{pp\pi}+V_{pp\sigma})^2 - \lambda_{\text{V}}^2 - \lambda_{\text{SOC}}^2\right],$$
$$\delta_2 = \text{Sign}\left[\frac{1}{4}(V_{pp\pi}+V_{pp\sigma})^2 - \lambda_{\text{SOC}}^2 - (\lambda_{\text{V}} - V_{pp\pi}+V_{pp\sigma})^2\right].$$

To simplify further these expressions, we note that when the product $V_{pp\sigma}V_{pp\pi} \leq 0$, the topological regime ($\delta_1\delta_2 < 0$) occurs whenever

$$\frac{9}{4}\left(\frac{V_{pp\sigma}+V_{pp\pi}}{\lambda_{\text{SOC}}}\right)^2 - \left(\frac{\lambda_{\text{V}}}{\lambda_{\text{SOC}}}\right)^2 > 1. \tag{S11}$$

*Parameters around the* $\Gamma$*-point*—In the following, we will be interested on modeling helical edge states propagating along zigzag boundaries, which exhibit a Dirac point close to the $\Gamma-$point, see Fig. S6a. Therefore, we need to adjust the Slater Koster parameters $V_{pp\sigma} = 2.0\,\text{eV}$ and $V_{pp\pi} = -0.21\,\text{eV}$ obtained around the $K$- and $K'$-points [S1]. Indeed, these parameters give rise to a bulk gap of $\sim 5$ eV around the $\Gamma$-point (see Fig. S6(b)), which is clearly in contrast to the one obtained using DFT calculations $\sim 1.5\,\text{eV}$ [S1, S2]. Thus, we have used instead $V_{pp\sigma} = 1.65\,\text{eV}$, and $V_{pp\pi} = -0.9\,\text{eV}$, which set the bulk gap in accordance to the DFT calculations, see Fig. S7a. In our calculations we model the topological regions using $\lambda_{\text{SOC}} = 0.435\,\text{eV}$, $\lambda_{\text{R}} = 0.032\,\text{eV}$, which are obtained by fitting DFT calculations around the $K$- and $K'$-points. Furthermore, we have used $\lambda_{\text{V}} = 0.3\,\text{eV}$ to place the Dirac crossing approximately at the same energy as the highest energy states of the valence band at $K$ and $K'$ points. This yields a Fermi velocity $\hbar v_{\text{F}} \approx 3.2\,\text{eV}\,\text{Å}$, which lies within the error-range of experimental fitting results in the main text.

### *2. Line defect*

During the process of growing bismuthene on SiC, the lattice develops line defects that distribute in the bulk of the material. Locally, the presence of these defects produces a change in the topological order. Thus, a pair of helical edge states emerge and propagate along them. Now, due to the small width of the topological defects $W \sim 2\,\text{nm}$, the helical edge states overlap, lifting the topological protection close to the Dirac point, where a gap $\Delta_{\text{DP}}$ opens. To have more insight on the low energy physics of the line defect, we make use of the tight-binding Bi/SiC Hamiltonian introduced in the previous section and define a trivial domain embedded between two topological regions. Here, the trivial domain is modeled by the set of Slater-Koster parameters $V_{pp\sigma}^{\text{DB}} = 0.6\,\text{eV}$, $V_{pp\pi}^{\text{DB}} = -0.45\,\text{eV}$, which clearly does not satisfy Eq. (S11). In Figs. S7a, b we compare the zigzag energy dispersion of a pure topological nanoribbon and one with an infinitely large (with periodic boundary conditions) line defect. As we advanced above, the nanoribbon with a trivial domain develops a gap of $\Delta_{\text{DP}} \sim 0.1\,\text{eV}$ at the Dirac point, see Fig. S7b. The size of the gap is controled by the width $W$ and by the Slater-Koster parameters $V_{pp\sigma}$ and $V_{pp\pi}$ set on the trivial region. These parameters tune the strength of the coupling between the orbitals of nearest neighbour atoms, see a similar scenario in Ref. S5.

Experimentally, the extension where the helical edges are coupled is bounded by the presence of kinks along the line defects, yielding finite strips of lengths up to $L \sim 25\,\text{nm}$. Thus, the resulting scenario consists basically of linear dispersion states confined in the defect, which exhibit the physics of a relativistic particle in a box, with quantized momentum $k = (n+1/2)\pi/L$ and energy $E = \hbar v_{\text{F}}(n+1/2)\pi/L$, where $n$ is an integer number and $L$ is the length of the box. In order to model this finite setup, we construct a flake Hamiltonian with a finite trivial region ($L$ and $W$) placed in the middle of a topological domain, see Fig. S7(c). It is important to note that we have removed spurious effects arising from the presence of topological armchair edges apearing at the outer part of the flake. For this reason, we have projected the Hamiltonian on a torus so we can remove the edge states from the areas away from the topological line defect, see Figs. S7c, d. The resulting discrete energy spectrum exhibits a constant energy difference of 0.1 eV and moreover, the local density of states integrated over the trivial region exhibits the typical standing wave pattern, showing an increasing number of modes as we go up in energy see Fig. S8. In addition, we show the probability density of the trivial region as a function resolved in position $x$ and $y$. Here, we can observe maxima at the edge between the trivial and topological regions decaying towards the domain boundary. This finite probability density inside the trivial domain is responsible for the gap opening at the Dirac point $\Delta_{\text{DP}}$.

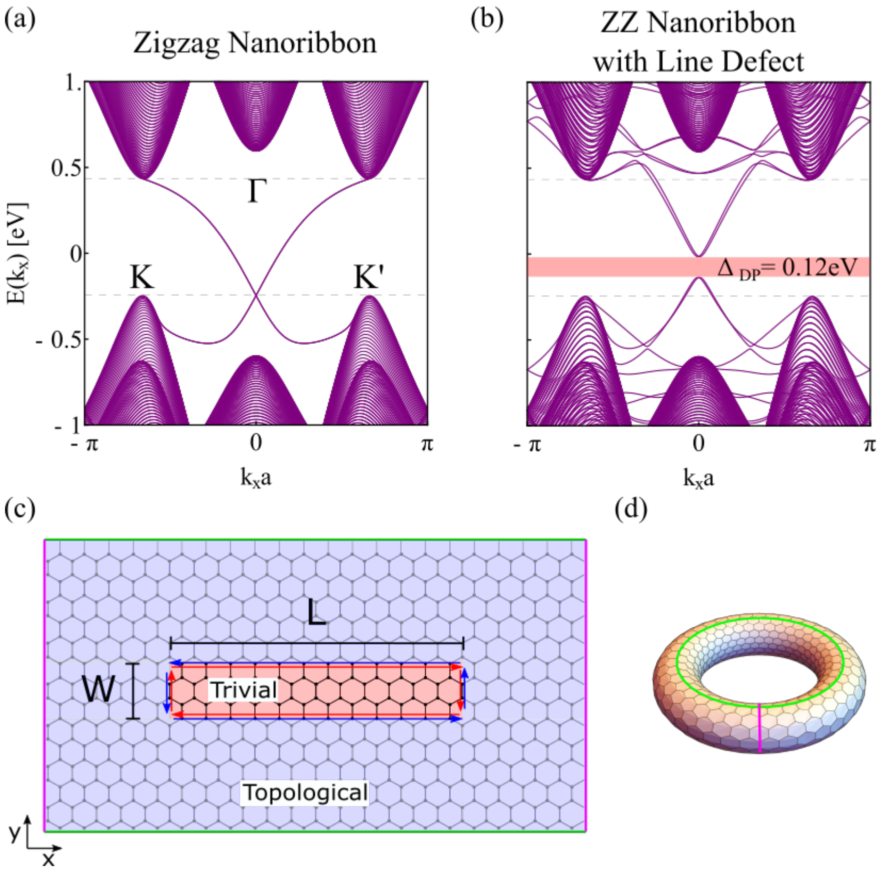


FIG. S7. Panel(a) and (b) show the ZZ nanoribbon spectrum without and with a topological line defect. In panel (b) a gap opens at the Dirac point $\Delta_{\rm DP} = 0.12$ eV. Panel (c) shows schematically the structure of Bismuthene with a topologically non-trivial (blue area) and trivial (red area) domains. The system features PBC in both directions, i.e. the 2D hexagonal lattice forms the surface of a torus as shown in (d). The red/blue arrows in (c) indicate spin down/up QSH edge modes moving in the directions of the arrows along the ZZ and AC edges.

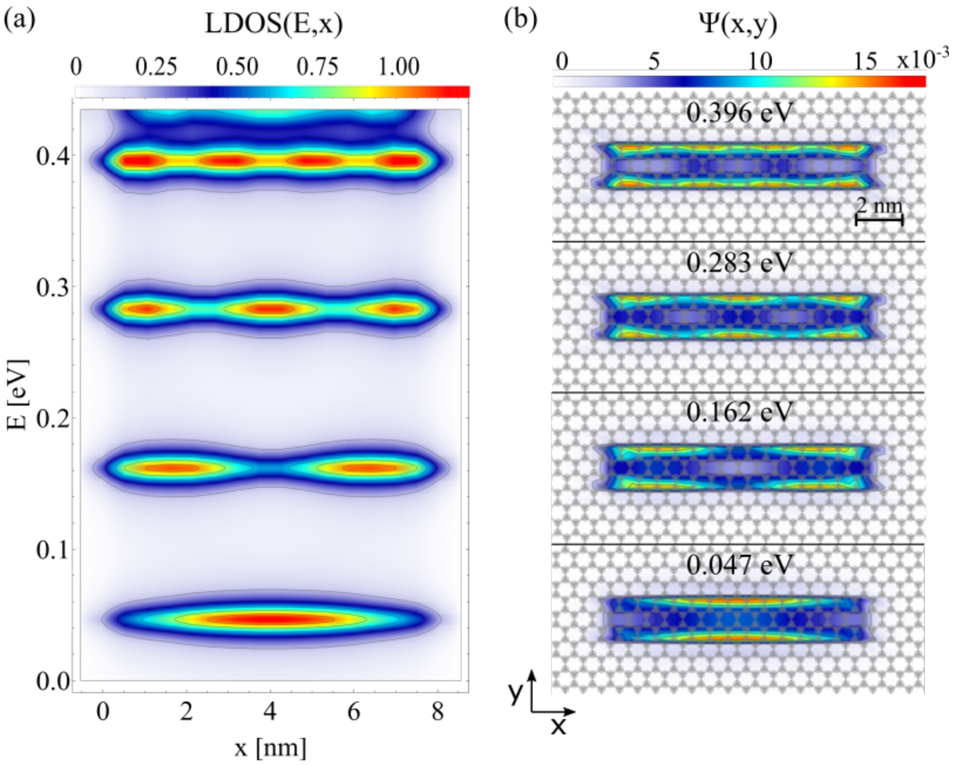


FIG. S8. In panel (a) we show the local density of states (LDOS) as a function of the longitudinal position $x$ of the DB, integrated over the width $W$ and energy. In panel (b) we show the probability density at the energies corresponding to the peaks in the LDOS as a function of $x$ and $y$.

| Fig. | $V_{\text{set}}$ [V] | $I_{\text{set}}$ [nA] | $T$ [K] |
|---|---|---|---|
| **a0** | -0.50 | 0.10 | 4.3 |
| **a1 − a2** | -1.00 | 0.10 | 4.3 |
| **b0** | -1.00 | 0.03 | 4.3 |
| **b1 − b3** | -0.80 | 0.10 | 4.3 |
| **c0** | -1.00 | 0.01 | 4.3 |
| **c1 − c2** | -1.00 | 0.30 | 4.3 |
| **d0** | -0.60 | 0.02 | 4.3 |
| **d1 − d3** | -0.60 | 0.20 | 4.3 |
| **e0** | -1.00 | 0.01 | 4.3 |
| **e1 − e4** | -1.00 | 0.30 | 4.3 |
| **f0** | -0.50 | 0.05 | 4.3 |
| **f1 − f4** | -0.60 | 0.50 | 4.3 |
| **g0** | -0.50 | 0.15 | 4.3 |
| **g1 − g4** | -0.50 | 0.15 | 4.3 |
| **h0** | -1.00 | 0.01 | 4.3 |
| **h1 − h4** | -0.80 | 0.30 | 4.3 |

TAB. S1. Scan parameters for DB segments a – h in Fig. S9a – h.

## G. Fabry-Pérot states in different domain boundaries

In order to demonstrate the ubiquitous and fully reproducible character of Fabry-Pérot states observed in bismuthene domain boundaries we have added the corresponding $\mathrm{d}I/\mathrm{d}V$ resonance data for eight more domain boundary segments in Figs. S9a – h. The depicted domain boundaries in the constant current STM images in Figs. S9a0 – h0 exhibit different lengths $L$ which are indicated inside the respective image. The $L$ values are estimated from the STM topographies (i.e., from the detected atomic structure) and hence have to be taken with some caution. Importantly, however, this uncertainty connected to $L$ does not enter in the in our evaluation of the edge state dispersion relation (presented in Fig. 4a of the paper), as we refrain from using the domain boundary lengths $L$ as reference points. Instead, in the main text we lay down how we employ an unbiased method, by directly inferring the spatial separation of modulation maxima $\lambda$ as indicated in the $\mathrm{d}I/\mathrm{d}V$ line profile of level E4 in Fig. 3g, thus avoiding any a priori assumptions on $L$.

The differential conductivity maps plotted above each constant current image are taken within the region of the respective domain boundary marked by the red rectangle in Figs. S9a0 – h0. Each differential conductivity map shows a Fabry-Pérot resonance with its respective energy with respect to $E_F$ indicated on the left side of each panel. Above each differential conductivity map we plot the differential conductivity line profiles resulting from integrating over the width of the domain boundary in the corresponding differential conductivity map. The arrows indicate the positions of resonance maxima, which can be obtained by fitting a Gaussian function in the vicinity of each resonance maximum (red dashed lines indicate best fits).

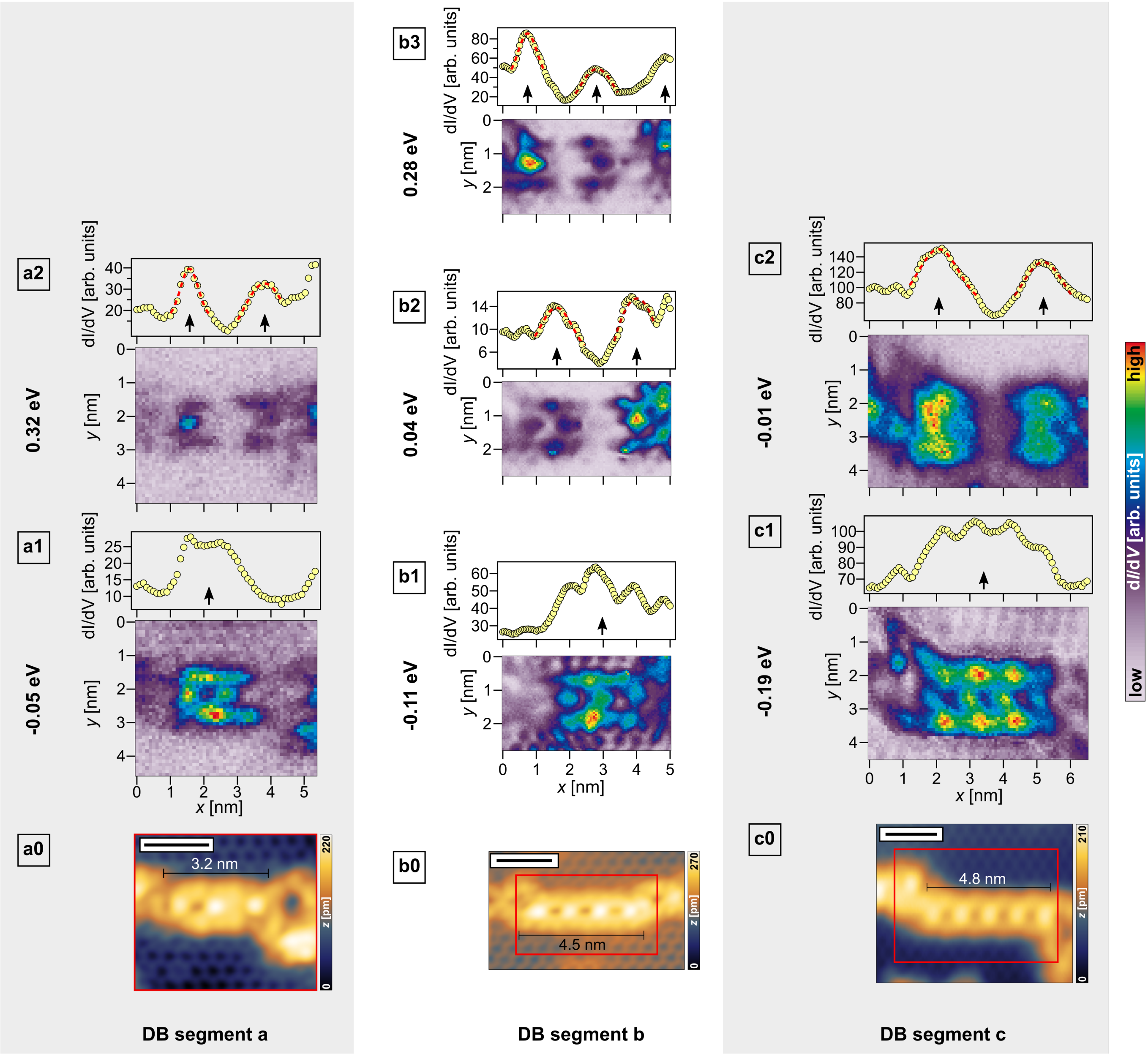


FIG. S9. **a – c, Fabry-Pérot states in different domain boundaries with varying lengths.** $\mathrm{d}I/\mathrm{d}V$ resonance data for eight domain boundary segments (see continuation of Fig. S9 in Figs. S9d – f, and S9g – h. **a0 – h0,** Constant current STM images of the domain boundaries. The length $L$ of each domain boundary is indicated inside the respective image. The $L$ values are estimated from the STM topographies (i.e., from the detected atomic structure) and are subject to an estimated uncertainty of $\approx \pm 0.5\,\mathrm{nm}$. The scale bar is 2 nm. **ai – hi, i $\geq$ 1,** The differential conductivity maps plotted above each constant current image are taken within the region of the respective domain boundary marked by the red rectangle in Figs. a0 – h0, and correspond to the i$^{\mathrm{th}}$ Fabry-Pérot resonance level. The respective energy with respect to $E_F$ is indicated on the left side of each panel. The differential conductivity line profiles resulting from integrating over the width of the domain boundary in the corresponding differential conductivity map are shown above each map. The arrows indicate the positions of resonance maxima, which are obtained by fitting the data in the vicinity of each resonance maximum with a Gaussian function (red dashed lines indicate best fits). Scan parameters are listed in Tab. S1.

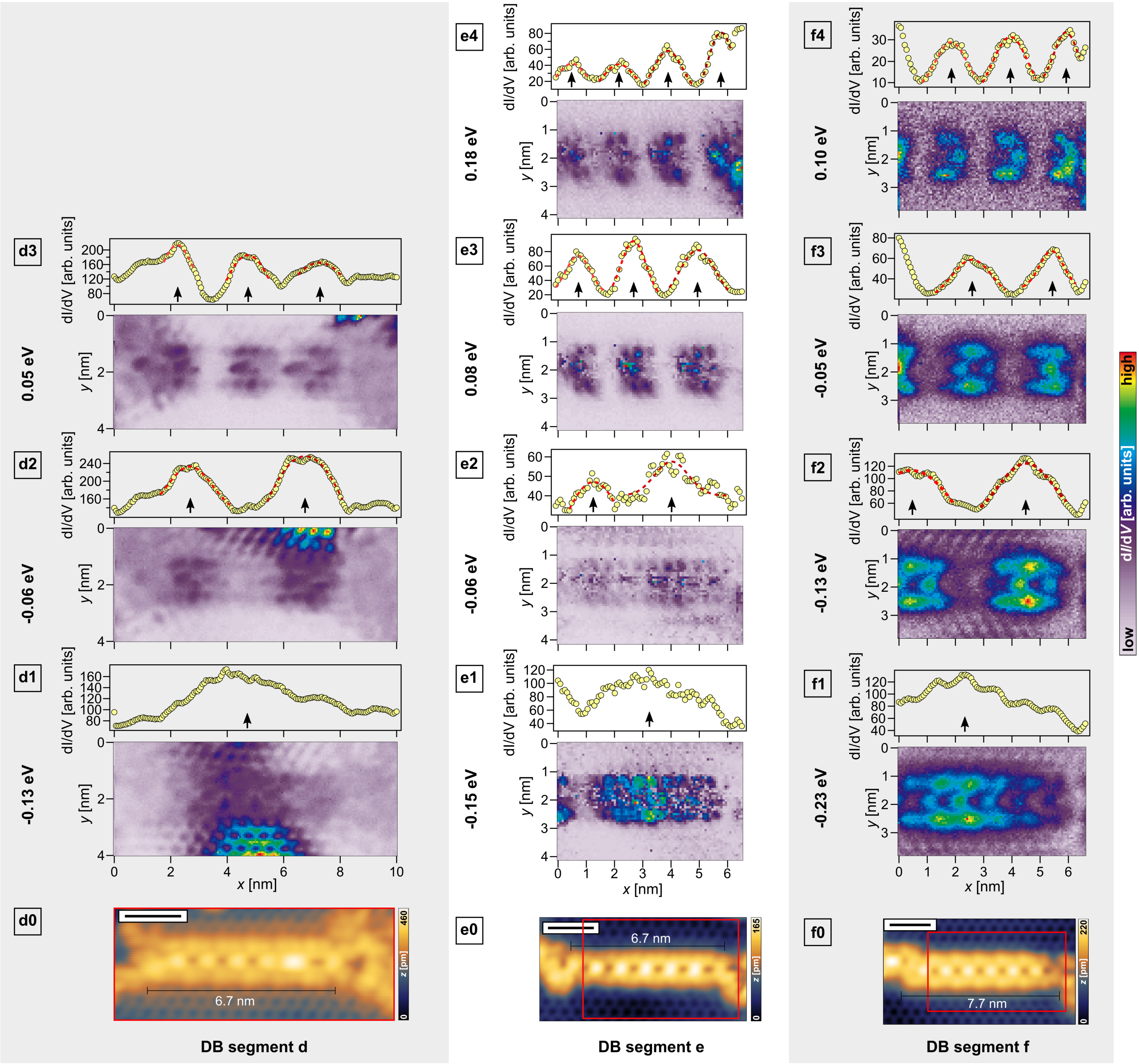


FIG. S9. **d – f, Fabry-Pérot states in different domain boundaries with varying lengths; continuation.**

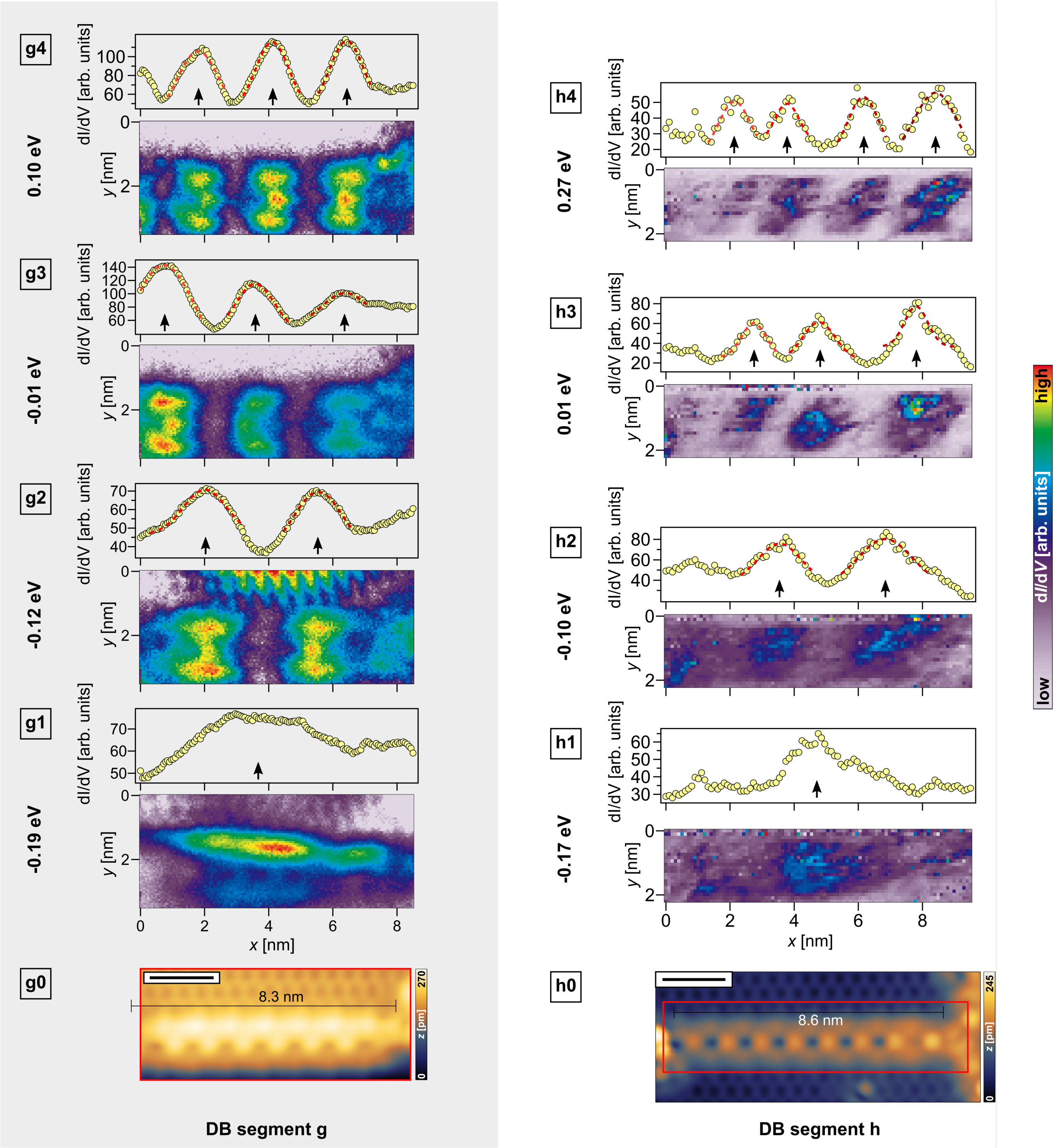


FIG. S9. **g – h, Fabry-Pérot states in different domain boundaries with varying lengths; continuation.**

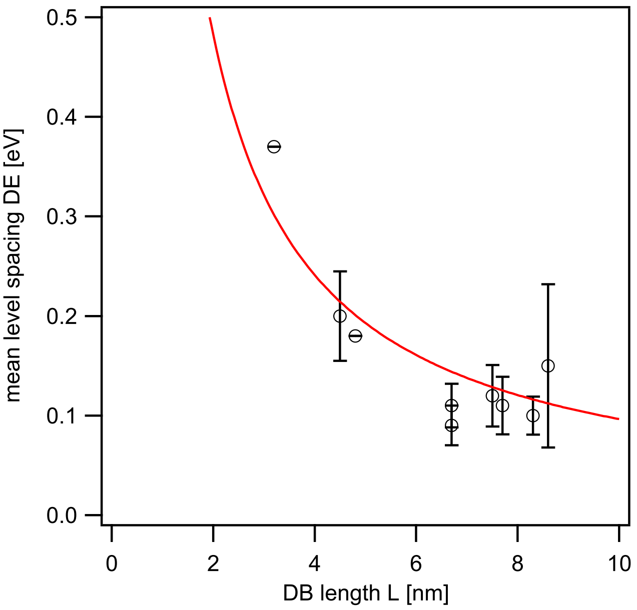


FIG. S10. Mean Fabry-Pérot resonance spacing $DE$ as a function of DB length $L$. The error bars indicate the std in the case of DBs, where more than two Fabry-Pérot resonances are observed. The red line indicates a $a/L$ function with $a = 0.965\,\mathrm{eV\,nm^{-1}}$.

### H. 1/L dependence of Fabry-Pérot resonances

In Fig. S10 we evaluate the mean energy spacing $DE$ of the Fabry-Pérot resonances shown in Fig. S9 and Fig. 3 as a function of the domain boundary length $L$. Indeed, it shows a systematic decrease of the level spacing roughly following an $a/L$ dependence (red curve). As we have mentioned above, there lies some uncertainty in the determination of the domain boundary length $L$, i.e. the resonator length, which is strengthened by the scattering centers (kinks or atomic defects) having also some finite extension.

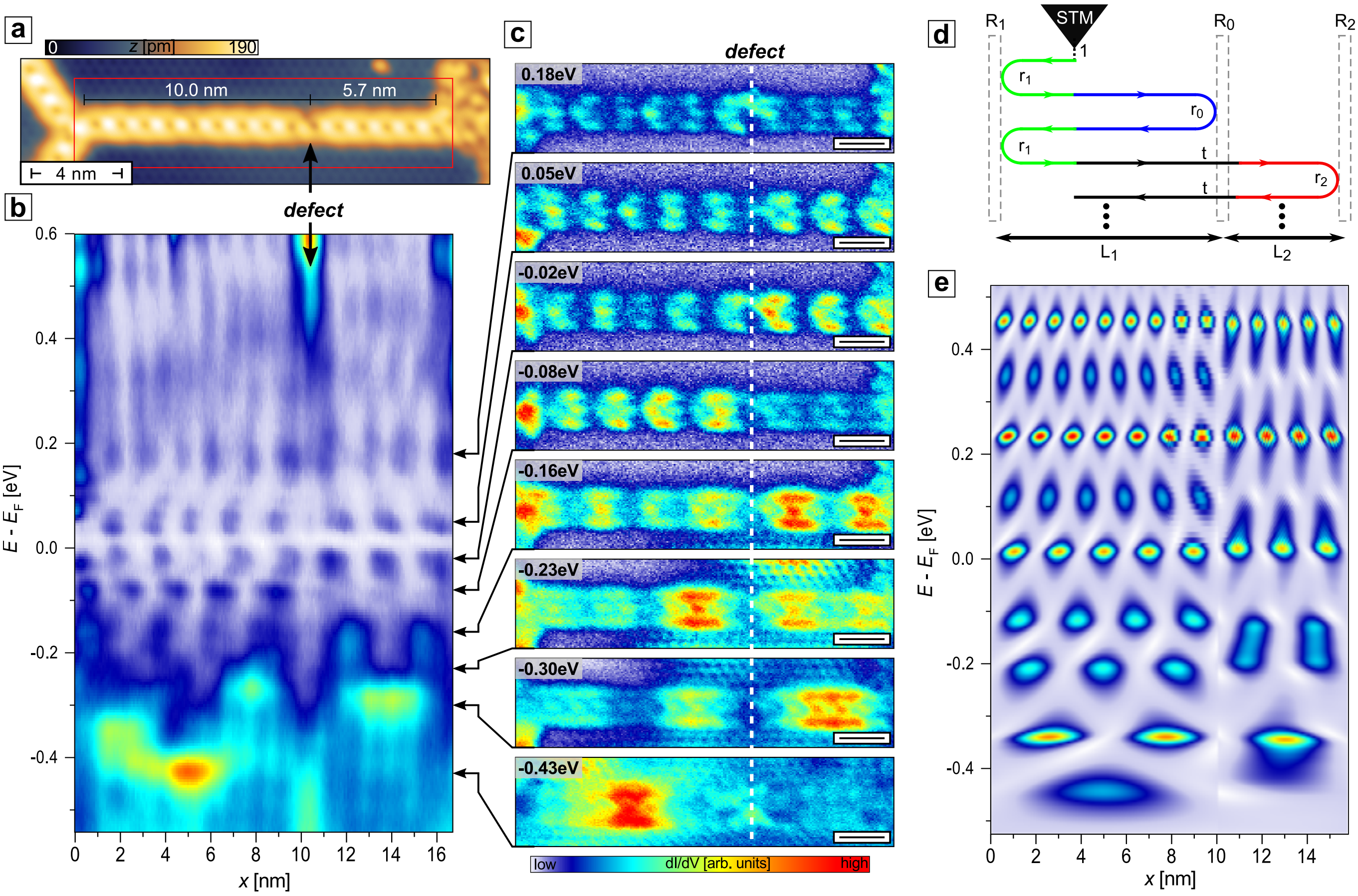


FIG. S11. **a**, Constant-current STM measurement of a DB with a defect inside, that splits the DB into two straight segments with different lengths. The red rectangle indicates the region for spatially resolved $\mathrm{d}I/\mathrm{d}V$ mapping. Scanning parameters: $V_{\mathrm{set}} = -1.2\,\mathrm{V}$, $I_{\mathrm{set}} = 10\,\mathrm{pA}$, $T = 4.35\,\mathrm{K}$. **b**, $\mathrm{d}I/\mathrm{d}V$ integrating over the width of the DB as a function of longitudinal position $x$. Energy dependent charge modulations are observed both in the left and right DB segment, for which towards higher energies their wavelengths shortens. The situation is reminiscent of a coupled double Fabry-Pérot resonator, see **d, e** for qualitative modelling. **c**, $\mathrm{d}I/\mathrm{d}V$ maps at constant energy marked with arrows in **b**. For certain energies the charge modulations are mainly confined within the left segment of the DB and for certain other energies mainly confined within the right segment of the DB. The scale bar is 2 nm. **d**, Schematic of coupled double Fabry-Pérot resonator for qualitative modelling of the observed charge modulations. The coupled resonator is composed of a first resonator with length $L1$ and a second resonator with length $L2$. Both resonators are coupled with a partially reflective wall $R_0$. Charge carriers injected into the resonator by the STM tip are reflected/transmitted multiple times at the resonators walls that are characterized by a reflectivity $R_i = |R_i|e^{i\arg(R_i)}$, where $i = 0, 1, 3$. In a plane wave scattering approach the electronic wave function $\psi$ at position $x$ can be modelled by summing over all possible scattering paths, see Eqs. (S12)–(S19) (the initial amplitude of the injected carrier is set to 1). **e,** Simulation of charge modulations in a coupled double Fabry-Pérot resonator in **d**. The false color plot shows $|\psi|^2$ from Eq. (S12) for model parameters: $L_1 = 10.0\,\mathrm{nm}$, $L_2 = 57.0\,\mathrm{nm}$, $R_0 = 0.7e^{i\pi}$, $R_1 = R_2 = 0.5e^{i\pi}$, and $E_{R/L} - E_F = \hbar v_F k_{R/L} + \mu$, where $\hbar v_F = 3.6\,\mathrm{eV\,Å}$ and $\mu = -0.56\,\mathrm{eV}$. The infinite sums in Eqs. (S13), (S14) were evaluated up to $j_F = 5$ and $m_F = 2$.

## I. Coupled Fabry-Pérot resonators

At the origin of referring to the observed charge modulations as "Fabry-Pérot resonances" instead of "quantum well states" lies the idea that the scattering centers exhibit a finite transmission probability, which implies that the coupled edge states at the domain boundary possess a finite probability of not being back-scattered. We exemplify this property for the special case of a coupled Fabry-Pérot resonator. This experimental situation is seen in the constant current image in Fig. S11a. This measurement shows a long domain boundary with a point defect inside. The left and the right segments have lengths $L_1$ and $L_2$, respectively, as indicated inside the image.

We conducted a full spatially resolved STS differential conductivity measurement within the region indicated by a red rectangle in Fig. S11a. The energy resolved and domain boundary width integrated $\mathrm{d}I/\mathrm{d}V$ spectrum is shown in Fig. S11b, whereas the spatially resolved $\mathrm{d}I/\mathrm{d}V$ maps at certain resonance energies are depicted in Fig. S11c. We first

concentrate on Fig. S11b: It can be clearly seen that charge modulations are present both to the left and to the right of the defect. It can also be seen that the modulations in the left segment, which is the longer one, set in beginning at lower energies and feature smaller energy separations compared to the modulations in the right segment, which is the shorter one. As already mentioned in the main text, the charge modulations can however be even better seen in the spatially resolved $\mathrm{d}I/\mathrm{d}V$ maps in Fig. S11c. The corresponding energies are indicated by the arrows pointing from Fig. S11c to the energy scale of Fig. S11b. Here the lowest image shows the charge modulation that corresponds to the first Fabry-Pérot resonance in the left segment. The $\mathrm{d}I/\mathrm{d}V$ in the right segment, on the other hand, is rather featureless at this energy. In the second-to-lowest panel in Fig. S11c the second Fabry-Pérot resonance is seen in the left segment, whereas the first Fabry-Pérot resonance is seen in the right segment. At higher energies, the subsequent Fabry-Pérot resonances appear both in the left and right segment.

For the qualitative interpretation of the data we introduce a coupled double Fabry-Pérot resonator model depicted in Fig. S11d. It is an extension to the model by Seo et al. [S6]. According to the observed topography of the domain boundary in Fig. S11a, the left resonator (1) has a length $L_1$ and the right resonator (2) a length $L_2$. The model exhibits a left, middle and right barrier depicted by gray dashed lines to simulate back-scattering off from point scatterers with finite reflectivities $R_p = |R_i|e^{i\arg(R_p)}$, with $p = 0, 1, 2$. In a plane wave scattering approach charge carriers with an energy-momentum dispersion $E(k_L, k_R)$, where $k_L, k_R$ describe the wave vector for left and right moving carriers, respectively, are injected from the STM tip at position $x$. They can experience reflection off or transmission through the barriers and eventually return to position $x$. The electronic wave function $\psi$ at position $x$ can be modelled by summing over all possible scattering paths. Setting the initial amplitude of the injected carrier to 1, we arrive at a series expansion for the electronic wave function $\psi$:

$$\psi = \begin{cases} \psi_1^{\mathrm{L}} + \psi_1^{\mathrm{R}} & -L_1 \leq x < 0 \\ \psi_2^{\mathrm{L}} + \psi_2^{\mathrm{R}} & 0 \leq x \leq L_2 \end{cases} \tag{S12}$$

$$\psi_1^{\mathrm{L}} = \sum_{j=0}^{\infty}\sum_{k=0}^{j}\sum_{n=0}^{k} \binom{j}{k}\binom{k}{n} \left[r_0^j + r_0^{j+1}\right] \times r_1^{j-k} \times t^k \times r_2^{k+n} \times R_0^n \tag{S13}$$

$$\psi_1^{\mathrm{R}} = 1 + \left(r_1 + t\sum_{m=0}^{\infty} r_2^{1+m} \times R_0^m\right)\psi_1^{\mathrm{L}} \tag{S14}$$

$$\psi_1^{\mathrm{L/R}} \mapsto \psi_2^{\mathrm{L/R}} : \{R_1 \leftrightarrow R_2,\, L_1 \leftrightarrow L_2,\, k_L \leftrightarrow k_R\}\ , \tag{S15}$$

where we have defined

$$r_0 := e^{i(k_L+k_R)(L_1-|x|)}R_1 \tag{S16}$$

$$r_1 := e^{i(k_L+k_R)|x|}R_0 \tag{S17}$$

$$t := t_R t_L := e^{i(k_L+k_R)|x|}\underbrace{\left[1 + |R_0|e^{i\arg(R_0)}\right]^2}_{T_0^2} \tag{S18}$$

$$r_2 := e^{i(k_L+k_R)L_2}R_2\,. \tag{S19}$$

Here $\psi_{1/2}^{\mathrm{L/R}}$ describe wave functions of initially left (L) and right (R) moving carriers in the first (1) and second (2) resonator.

The above modelling assumes a complex reflectively $R = |R|e^{i\arg(R)}$, whose amplitude will generally be less than unity ($|R| < 1$). It therefore expands the qualitative explanation of the observed Fabry-Pérot-like charge modulations by taking into account that the edge state electrons are not perfectly reflected by the scattering centers, just as in an optical Fabry-Pérot interferometer. Although, microscopic details of $R$ could only be inferred from ab-initio atomistic calculations (which is not addressed here), capturing the respective scattering potential, already our qualitative modeling ($|R| = const. < 1$ and $\arg(R) = \pi$) is able to explain characteristic features of the measured $\mathrm{d}I/\mathrm{d}V$ spectra: (1) a rather smooth background signal associated with the unscattered, i.e., transmitted part of the propagating edge state, and (2) the strongly modulated (along the domain boundary, Figs. 3f, g) and peaky (in energy, Fig. 3d) component resulting from the backscattering-induced interference. For a qualitative modelling of the data in Figs. S11b, c that reflects the local density of states (LDOS) in the system, we used the following parameters for the calculation of $|\psi|^2 \propto$ LDOS in Fig. S11d: $L_1 = 10.0\,\mathrm{nm}$, $L_2 = 5.7\,\mathrm{nm}$, $R_0 = 0.7e^{i\pi}$, $R_1 = R_2 = 0.5e^{i\pi}$, and $E_{R/L} - E_F = \hbar v_F k_{R/L} + \mu$, where $\hbar v_F = 3.6\,\mathrm{eV\,Å}$ and $\mu = -0.56\,\mathrm{eV}$. The infinite sums in Eqs. (S13), (S14) were

evaluated up to $j_F = 5$ and $m_F = 2$.


[S1] Reis, F. *et al.* Bismuthene on a SiC substrate: A candidate for a high-temperature quantum spin Hall material. *Science* **357**, 287–290 (2017).

[S2] Dominguez, F. *et al.* Testing topological protection of edge states in hexagonal quantum spin Hall candidate materials. *Phys. Rev. B* **98**, 161407 (2018). URL https://link.aps.org/doi/10.1103/PhysRevB.98.161407.

[S3] Li, G. *et al.* Theoretical paradigm for the quantum spin Hall effect at high temperatures. *Phys. Rev. B* **98**, 165146 (2018). URL https://link.aps.org/doi/10.1103/PhysRevB.98.165146.

[S4] Fu, L. & Kane, C. L. Topological insulators with inversion symmetry. *Phys. Rev. B* **76**, 045302 (2007). URL https://link.aps.org/doi/10.1103/PhysRevB.76.045302.

[S5] Lima, E. N., Schmidt, T. & Nunes, R. W. Topologically Protected Metallic States Induced by a One-Dimensional Extended Defect in the Bulk of a 2D Topological Insulator. *Nano Lett.* **16**, 4025–4031 (2016).

[S6] Seo, J. *et al.* Transmission of topological surface states through surface barriers. *Nature* **466**, 343–346 (2010).
